# Genetic Variants in MicroRNA Machinery Genes Are Associate with Idiopathic Recurrent Pregnancy Loss Risk

**DOI:** 10.1371/journal.pone.0095803

**Published:** 2014-04-25

**Authors:** Yong Wook Jung, Young Joo Jeon, HyungChul Rah, Ji Hyang Kim, Ji Eun Shin, Dong Hee Choi, Sun Hee Cha, Nam Keun Kim

**Affiliations:** 1 Department of Obstetrics and Gynecology, CHA Gangnam Medical Center, CHA University, Seoul, South Korea; 2 Institute for Clinical Research, CHA Bundang Medical Center, CHA University, Seongnam-si, South Korea; 3 Department of Obstetrics and Gynecology, CHA Bundang Medical Center, CHA University, Seongnam-si, South Korea; Baylor College of Medicine, United States of America

## Abstract

**Objective:**

Key molecules involved in microRNA (miRNA) biogenesis, such as DROSHA, XPO5, and DICER, have been identified in trophoblast cells, confirming that the miRNA biogenesis pathway is active in human placenta. In addition, miRNAs regulate uterine gene expression associated with inflammatory responses during the peri-implantation period and participate in maternal-fetal immune tolerance. The purpose of this study was to demonstrate whether genetic polymorphisms in miRNA machinery genes show an association with idiopathic recurrent pregnancy loss (RPL) in Korean women.

**Study design:**

We performed a case-control study with 238 controls and 338 women who had experienced at least two consecutive pregnancy losses between 1999 and 2010. Genotypes of miRNA machinery genes, including *DICER* rs3742330, *DROSHA* rs10719, RAN GTPase (*RAN*) rs14035, and exportin-5 (*XPO5*) rs11077 were analyzed by polymerase chain reaction-restriction fragment length polymorphism (PCR-RFLP) assay. The logistic odds ratios (ORs) of RPL were estimated with a 95% confidence interval (CI) in multivariate analysis after maternal age adjustment. Gene-gene interactions among the loci of the four gene polymorphisms were evaluated using the multifactor dimensionality reduction (MDR) method.

**Results:**

The *RAN* rs14035 CC genotype and *DICER* rs3742330/*DROSHA* rs10719 GG/TC+CC, rs3742330/*RAN* rs14035 GG/CC, and *DICER* rs3742330/*XPO5* rs11077 GG/AC+CC combinations were significantly associated with increased RPL risk, whereas the *RAN* rs14035 CT, *DICER* rs3742330/*RAN* rs14035 AA+AG/CT+TT, *DROSHA* rs10719/*RAN* rs14035 TC+CC/CT+TT, and *RAN* rs14035/*XPO5* rs11077 CT+TT/AA combinations reduced RPL risk. The A-T-T-C and G-C-T-A allele combinations (DICER/DROSHA/RAN/XPO5) were 20 times more frequent in the RPL group than in the control group.

**Conclusion:**

Our study demonstrates the relationship between RPL development and the polymorphism of the miRNA machinery gene *RAN* and combined genotype of *DROSHA*/*DICER*.

## Introduction

The incidence of spontaneous pregnancy loss has been estimated to be 30% [1]. However, less than 5% of women will experience two or more consecutive pregnancy losses [2]. Therefore, the loss of two or more clinically recognized pregnancies is considered a distinct disorder and defined as recurrent pregnancy loss (RPL) for additional evaluation and treatment [3]. Despite the various etiologies that result in RPL, including cytogenetic abnormalities, antiphospholipid syndrome, uterine anomalies, hereditary thrombophilia, autoimmunity, sperm quality, and environmental factors, the cause of RPL still remains undetermined in the majority of cases.

MicroRNAs (miRNAs) are ∼19–25-nucleotide single-strand non-coding RNA species that induce post-transcriptional gene silencing and mediate translational repression through binding to target mRNA, leading to subsequent mRNA degradation. The recent elucidation of miRNA function has provided new insight into the regulation of gene expression. Key molecules involved in miRNA biogenesis, such as DROSHA, XPO5, and DICER, have been identified in trophoblast cells, confirming that the miRNA biogenesis pathway is active in human placenta [4], [5]. It has been demonstrated that the human placenta produces a large number of miRNAs and that those are involved in placental development [6]. In addition, miRNAs regulate uterine gene expression associated with inflammatory responses during the peri-implantation period and participate in maternal-fetal immune tolerance [7], [8]. There are numerous reports that demonstrate the association of aberrant miRNA expression with various human diseases related to reproductive conditions [9]–[13]. Several studies have reported that genetic polymorphisms are associated with RPL development [14]–[17]. Furthermore, recent studies have found that nucleotide variations within pri-miRNA molecules affect miRNA processing and result in altered miRNA expression and that a single-nucleotide polymorphism (SNP) in a certain miRNA is associated with litter size in pigs [18], [19].

Although SNPs have been widely implicated in RPL development, such evidence is lacking for miRNA biogenesis pathway genes. This study was performed to investigate whether polymorphisms in the miRNA machinery genes *DROSHA* (rs10719), *DICER* (rs3742330), *RAN* (rs14035), and *XPO5* (rs11077) are associated with the prevalence of RPL in Korean women. Our previous study investigated the allele frequencies of nine SNPs in those four essential genes in primary ovarian insufficiency and control subjects [20]. The results revealed that some polymorphic sites, such as *XPO5* rs3324334 and *XPO5* rs11544382 that had been reported to be polymorphic markers, were monomorphic in all Korean subjects that we genotyped. Therefore, we examined the remaining four polymorphic sites in those four genes.

## Materials and Methods

### Study participants

The study was reviewed and approved by the institutional review board (IRB) of CHA Bundang Medical Center in 1999, and written informed consent was obtained from all participants. IRB approved this consent procedure. The study population consisted of Korean participants (Asian) recruited from the Department of Obstetrics and Gynecology of CHA Bundang Medical Center, CHA University (Seongnam-si, South Korea) between March 1999 and February 2010. Participants with a history of smoking, alcohol abuse, cancer, radiation exposure, autoimmune disorder, genetic syndromes, or systemic disease affecting ovarian function, such as diabetes mellitus, were excluded from the study based on medical history and physical examination. Pregnancy loss was diagnosed with hCG testing, ultrasound, and/or physical examination before 20 weeks of gestational age. RPL was defined as two or more consecutive pregnancy losses before 20 weeks of gestational age. All participants in the RPL group experienced recurrent miscarriage with the same partner. RPL patients with previous live births were excluded from this study.

To diagnose idiopathic RPL, we adopted a set of exclusion criteria for the study group. Patients who were diagnosed with RPL due to anatomic, chromosomal, hormonal, infectious, autoimmune, or thrombotic causes were excluded from the study group. To identify anatomic abnormalities in RPL patients, sonography, hysterosalpingogram, hysteroscopy, computerized tomography, and magnetic resonance imaging were used. Karyotyping was performed with standard protocols [21]. Hormonal causes included hyperprolactinemia, luteal insufficiency, and thyroid disease and were evaluated by measuring levels of prolactin, thyroid-stimulating hormone, free T4, follicle-stimulating hormone, luteinizing hormone, and progesterone in peripheral blood. Lupus anticoagulant and anticardiolipin antibodies were examined for autoimmune causes such as lupus and antiphospholipid syndrome. Thrombotic causes were defined as thrombophilia and were evaluated by deficiencies of protein C and protein S and by the presence of anti-β2 glycoprotein antibody. The study group consisted of 338 women diagnosed with idiopathic RPL [age range, 23–43 years; mean age ± standard deviation (SD), 32.81±4.33 years] (Table S1).

The enrollment criteria for the control group included regular menstrual cycles, normal karyotype of 46XX, a history of at least one naturally conceived pregnancy, and no history of pregnancy loss. The control group was comprised of 238 women [age range, 22–45 years; mean age ± SD, 33.38±5.79 years]. The average gestational age and number of pregnancy losses in the RPL group were 7.32±1.85 weeks and 3.04±1.61 losses, respectively.

### Genotyping

Peripheral blood samples were collected for genotyping. Genomic DNA was extracted from collected blood in the presence of anticoagulant using a G-DEX blood extraction kit (iNtRON Biotechnology, Seongnam-si, Korea). Nucleotide changes were determined by polymerase chain reaction-restriction fragment length polymorphism (PCR-RFLP) analysis. The PCR primers for this study are shown in Table S2. Restriction enzyme digestion was carried out using the following enzymes and conditions: *Nla*III (*DROSHA* rs10719) (New England BioLabs, Ipswich, MA), *Ban*I (*DICER* rs3742330), and *Eco*RI (*XPO5* rs11077) at 37°C for 16 hours and *Bsl*I (*RAN* rs14035) at 55°C for 16 hours. Genotypes determined by RFLP analysis were confirmed by two independent investigators. We confirmed genotypes again by sequencing 10% of the samples by random selection.

### Statistical analysis

The differences in genotype and allele combination frequencies between idiopathic RPL subjects and controls were compared using the multivariate logistic regression and Fisher's exact test, respectively. Allele frequencies were calculated to identify deviations from Hardy–Weinberg equilibrium (HWE) using *P* = .05 as a threshold. Odds ratios (ORs), adjusted odds ratios (AORs), and 95% confidence intervals (CIs) were used to measure the strength of association between genotypes and idiopathic RPL. Two-tailed *P* values <.05 were considered statistically significant. The ORs were adjusted by the age of the participants because maternal age is a known risk factor for spontaneous abortion and RPL [22].

Gene-gene interactions among the SNP loci were analyzed using the log-linear model-based multifactor dimensionality reduction (LM-MDR) and MDR software version 2.0 (available at www.epistasis.org) [23]–[25]. The allele combination frequencies for the selected models by MDR analysis were estimated with the HAPSTAT program version 3.0 (www.bios.unc.edu/~lin/hapstat). The false-positive discovery rate (FDR) correction was used to adjust multiple comparison tests, and associations with FDR-adjusted *P* value <.05 were considered to be significant data. Statistical analyses were performed with GraphPad Prism 4.0 (GraphPad Software Inc., San Diego, CA) and StatsDirect software version 2.4.4 (StatsDirect Ltd., Altrincham, UK). The statistical power was calculated using G*POWER 3.9.1 (Institut für Psychologie, Christian-Albrechts-Universität Kiel, Kiel, Germany).

## Results

The genotypic distribution and allele frequencies of *DROSHA* rs10719, *DICER1* rs3742330, *RAN* rs14035, and *XPO5* rs11077 are shown in Table 1. All of the genes that were analyzed showed polymorphisms and occurred in HWE in both groups. The *DICER* rs3742330, *DROSHA* rs10719, and *XPO5* rs11077 polymorphisms were not associated with the prevalence of RPL. However, the *RAN* rs14035 polymorphism was associated with the prevalence of RPL. The frequency of the CT genotype of *RAN* rs14035 was significantly higher in control subjects than in RPL patients. This association remained statistically significant after FDR correction. Thus, *RAN* rs14035 CT+TT was associated with the decreased prevalence of RPL (AOR = 0.657, 95% CI = 0.469–0.921).

**Table 1:** Comparison of genotype frequencies of polymorphisms in miRNA machinery genes between RPL and control subjects.

Characteristics	Controls (n = 238)	RPL patients (n = 338)	AOR (95% CI)	*P* a	*P* b	Statistical power (%)
***DICER*** ** rs3742330**						
AA	75 (31.5)	119 (35.2)	1.000 (reference)			
AG	123 (51.7)	152 (45.0)	0.741 (0.510–1.077)	0.117	0.234	42.5
GG	35 (14.7)	67 (19.8)	1.196 (0.724–1.975)	0.484	0.776	12.5
Dominant (AA vs. AG+GG)			0.841 (0.590–1.198)	0.336	0.672	15.4
Recessive (AA+AG vs. GG)			1.428 (0.913–2.235)	0.119	0.476	48.4
HWE *P*	0.18	0.147				
***DROSHA*** ** rs10719**						
TT	110 (46.2)	161 (47.6)	1.000 (reference)			
TC	108 (45.4)	150 (44.4)	0.943 (0.667–1.335)	0.742	0.977	6.4
CC	20 (8.4)	27 (8.0)	0.919 (0.487–1.733)	0.794	0.794	5.6
Dominant (TT vs. TC+CC)			0.933 (0.669–1.303)	0.685	0.859	7.0
Recessive (TT+TC vs. CC)			0.907 (0.494–1.665)	0.752	0.954	6.4
HWE *P*	0.363	0.329				
***RAN*** ** rs14035**						
CC	123 (51.7)	210 (62.1)	1.000 (reference)			
CT	104 (43.7)	113 (33.4)	0.640 (0.452–0.906)	0.012	0.048	72.6
TT	11 (4.6)	15 (4.4)	0.796 (0.354–1.791)	0.582	0.776	72.5
Dominant (CC vs. CT+TT)			0.657 (0.469–0.921)	0.015	0.060	70.0
Recessive (CC+CT vs. TT)			0.977 (0.439–2.172)	0.954	0.954	4.6
HWE *P*	0.059	0.967				
***XPO5*** ** rs11077**						
AA	197 (82.8)	279 (82.5)	1.000 (reference)			
AC	39 (16.4)	53 (15.7)	0.993 (0.632–1.561)	0.977	0.977	50.5
CC	2 (0.8)	6 (1.8)	1.896 (0.357–10.061)	0.453	0.776	12.6
Dominant (AA vs. AC+CC)			1.041 (0.670–1.617)	0.859	0.859	53.4
Recessive (AA+AC vs. CC)			1.911 (0.360–10.140)	0.447	0.894	2.7
HWE *P*	0.964	0.072				

Note: For AOR, OR was adjusted by age of participants. RPL = recurrent pregnancy loss; AOR = adjusted odds ratio; CI = confidence interval; HWE = Hardy–Weinberg equilibrium;

a Fisher's exact test;

b FDR-adjusted *P* value.

The genotype frequencies were stratified according to the number of consecutive pregnancy losses (Table S3). For *DROSHA* rs10719 and *XPO5* rs11077, there were no statistically significant differences between the two groups. The *DICER* rs3742330 recessive model (GG compared with AA+AG) was associated with subjects who had history of three or more and four or more pregnancy losses (AOR = 1.718, 95% CI = 1.032–2.861 and AOR = 2.170, 95% CI = 1.181–3.986, respectively), For *RAN* rs14035, the CT genotype was less frequently observed in the subgroups of RPL participants with two or more and four or more consecutive pregnancy losses (AOR = 0.624, 95% CI = 0.412–0.947 and AOR = 0.502, 95% CI = 0.287–0.879, respectively).

We performed combination analyses for miRNA machinery gene polymorphisms that we tested for the study (Table 2). These analyses revealed that the *DICER* rs3742330 GG/*DROSHA* rs10719 TC+CC genotype was significantly more frequent in RPL patients than in control subjects (AOR = 1.990, 95% CI = 1.048–3.778). The frequency of the *DICER* rs3742330 AA+AG/*RAN* rs14035 CT+TT genotype was significantly higher in the control group (AOR = 0.688, 95% CI = 0.475–0.997). In addition, *DROSHA* rs10719 TC+CC/*RAN* rs14035 CT+TT and *RAN* rs14035 CT+TT/*XPO5* rs11077 AA genotypes were more frequently observed in control participants (AOR = 0.621, 95% CI = 0.389–0.991, AOR = 0.631, 95% CI = 0.435–0.914, respectively). Combination analysis according to the number of pregnancy losses was also performed (Table 3). Although these associations with RPL were not significant after FDR correction in combination analysis (Table 2), those associations became more evident when we performed combination analysis according to the number of pregnancy losses even after FDR correction (Table 3). In this combination analysis, there was a significant association between RPL development and the *DICER/DROSHA* GG/TC+CC, *DICER/RAN* GG/CC, and *DICER/XPO5* GG/AC+CC genotypes. The *RAN* CT+TT genotype showed a significant association with RPL when it was combined with the *XPO5* AA genotype. These significances were maintained when we performed multiple comparison correction using FDR, for all except the *RAN/XPO5* CT+TT/AA genotype. Thus, both individual and combined genotyping analysis suggested that both the *DICER* rs3742330 GG and *RAN* rs14035 CC genotypes are associated with RPL prevalence.

**Table 2:** Combination analysis of polymorphisms of miRNA machinery genes in RPL patients and control participants.

Genotypes	Controls (n = 238)	RPL patients (n = 338)	AOR (95% CI)	*P* a	*P* b	Statistical power (%)
***DICER/DROSHA***						
AA+AG/TT	90 (37.8)	141 (41.7)	1.000 (reference)			
AA+AG/TC+CC	113 (47.5)	130 (38.5)	0.729 (0.505–1.052)	0.091	0.273	45.7
GG/TT	20 (8.4)	20 (5.9)	0.633 (0.322–1.245)	0.185	0.370	25.5
GG/TC+CC	15 (6.3)	47 (13.9)	1.990 (1.048–3.778)	0.036	0.138	64.0
***DICER/RAN***						
AA+AG/CC	107 (45.0)	168 (49.7)	1.000 (reference)			
AA+AG/CT+TT	96 (40.3)	103 (30.5)	0.688 (0.475–0.997)	0.048	0.273	56.5
GG/CC	16 (6.7)	42 (12.4)	1.688 (0.903–3.156)	0.101	0.303	42.0
GG/CT+TT	19 (8.0)	25 (7.4)	0.826 (0.433–1.576)	0.562	0.674	8.4
***DICER/XPO5***						
AA+AG/AA	167 (70.2)	226 (66.9)	1.000 (reference)			
AA+AG/AC+CC	36 (15.1)	45 (13.3)	0.963 (0.592–1.566)	0.878	0.905	5.3
GG/AA	30 (12.6)	53 (15.7)	1.308 (0.801–2.137)	0.283	0.425	20.4
GG/AC+CC	5 (2.1)	14 (4.1)	2.055 (0.725–5.822)	0.175	0.350	32.9
***DROSHA/RAN***						
TT/CC	60 (25.2)	102 (30.2)	1.000 (reference)			
TT/CT+TT	50 (21.0)	59 (17.5)	0.694 (0.424–1.137)	0.147	0.294	37.6
TC+CC/CC	63 (26.5)	108 (32.0)	1.024 (0.654–1.604)	0.917	0.917	5.2
TC+CC/CT+TT	65 (27.3)	69 (20.4)	0.621 (0.389–0.991)	0.046	0.138	65.0
***DROSHA/XPO5***						
TT/AA	90 (37.8)	133 (39.3)	1.000 (reference)			
TT/AC+CC	20 (8.4)	28 (8.3)	0.945 (0.502–1.781)	0.861	0.905	5.3
TC+CC/AA	107 (45.0)	146 (43.2)	0.914 (0.633–1.319)	0.631	0.757	73.3
TC+CC/AC+CC	21 (8.8)	31 (9.2)	1.016 (0.548–1.886)	0.959	0.959	5.0
***RAN/XPO5***						
CC/AA	103 (43.3)	177 (52.4)	1.000 (reference)			
CC/AC+CC	20 (8.4)	33 (9.8)	0.964 (0.525–1.768)	0.905	0.905	5.1
CT+TT/AA	94 (39.5)	102 (30.2)	0.631 (0.435–0.914)	0.015	0.090	73.3
T+TT/AC+CC	21 (8.8)	26 (7.7)	0.708 (0.376–1.334)	0.285	0.428	17.7

Note: For AOR, OR was adjusted by age of participants. RPL = recurrent pregnancy loss; AOR = adjusted odds ratio; CI = confidence interval.

a Fisher's exact test;

b FDR-adjusted *P* value.

**Table 3:** Combination analysis of polymorphisms of miRNA machinery genes in RPL patients and control subjects according to the number of previous pregnancy losses.

Genotypes	Controls (n = 238)	PL = 2 (n = 173)	AOR (95% CI)	*P* a	*P* b	Statistical power (%)	PL≥3 (n = 165)	AOR (95% CI)	*P* a	*P* b	Statistical power (%)	PL≥4 (n = 81)	AOR (95% CI)	*P* a	*P* b	Statistical power (%)
***DICER/DROSHA***																
AA+AG/TT	90 (37.8)	77 (44.5)	1.000 (reference)				64 (38.8)	1.000 (reference)				25 (30.9)	1.000 (reference)			
AA+AG/TC+CC	113 (47.5)	67 (38.7)	0.705 (0.458–1.086)	0.113	0.170	53.7	63 (38.2)	0.767 (0.492–1.196)	0.242	0.363	52.6	34 (42.0)	1.083 (0.603–1.946)	0.789	0.871	38.7
GG/TT	20 (8.4)	8 (4.6)	0.478 (0.199–1.148)	0.099	0.170	58.5	12 (7.3)	0.815 (0.371–1.789)	0.610	0.610	13.5	6 (7.4)	1.088 (0.393–3.009)	0.871	0.871	6.3
GG/TC+CC	15 (6.3)	21 (12.1)	1.596 (0.765–3.327)	0.213	0.213	33.6	26 (15.8)	2.460 (1.202–5.036)	0.014	0.042	82.9	16 (19.8)	4.084 (1.752–9.520)	0.001	0.003	97.2
***DICER/RAN***																
AA+AG/CC	107 (45.0)	91 (52.6)	1.000 (reference)				77 (46.7)	1.000 (reference)				38 (46.9)	1.000 (reference)			
AA+AG/CT+TT	96 (40.3)	53 (30.6)	0.672 (0.433–1.043)	0.077	0.231	60.5	50 (30.3)	0.721 (0.459–1.130)	0.154	0.231	46.4	21 (25.9)	0.615 (0.336–1.124)	0.114	0.171	56.9
GG/CC	16 (6.7)	18 (10.4)	1.330 (0.641–2.763)	0.444	0.444	17.8	24 (14.5)	2.095 (1.041–4.220)	0.038	0.114	68.0	15 (18.5)	2.731 (1.223–6.098)	0.014	0.042	75.3
GG/CT+TT	19 (8.0)	11 (6.4)	0.687 (0.311–1.520)	0.354	0.444	27.0	14 (8.5)	0.996 (0.467–2.121)	0.991	0.991	5.2	7 (8.6)	1.035 (0.400– 2.681)	0.943	0.943	5.0
***DICER/XPO5***																
AA+AG/AA	167 (70.2)	123 (71.1)	1.000 (reference)				103 (62.4)	1.000 (reference)				50 (61.7)	1.000 (reference)			
AA+AG/AC+CC	36 (15.1)	21 (12.1)	0.834 (0.462–1.508)	0.549	0.762	16.0	24 (14.5)	1.113 (0.624–1.982)	0.718	0.718	10.4	9 (11.1)	0.838 (0.375–1.872)	0.666	0.666	13.4
GG/AA	30 (12.6)	24 (13.9)	1.095 (0.610–1.967)	0.762	0.762	8.9	29 (17.6)	1.552 (0.881–2.733)	0.128	0.192	46.2	15 (18.5)	1.671 (0.833–3.354)	0.148	0.222	40.3
GG/AC+CC	5 (2.1)	5 (2.9)	1.277 (0.359–4.537)	0.706	0.762	9.0	9 (5.5)	2.909 (0.948–8.925)	0.062	0.186	61.0	7 (8.6)	4.661 (1.417–15.330)	0.011	0.033	75.5
***DROSHA/RAN***																
TT/CC	60 (25.2)	54 (31.2)	1.000 (reference)				48 (29.1)	1.000 (reference)				21 (25.9)	1.000 (reference)			
TT/CT+TT	50 (21.0)	31 (17.9)	0.706 (0.394–1.262)	0.240	0.360	38.8	28 (17.0)	0.685 (0.377–1.248)	0.216	0.324	42.2	10 (12.3)	0.553 (0.236–1.296)	0.173	0.309	55.0
TC+CC/CC	63 (26.5)	55 (31.8)	0.983 (0.585–1.651)	0.947	0.947	5.9	53 (32.1)	1.087 (0.638–1.851)	0.760	0.760	10.0	32 (39.5)	1.534 (0.790–2.975)	0.206	0.309	44.7
TC+CC/CT+TT	65 (27.3)	33 (19.1)	0.587 (0.335–1.029)	0.063	0.189	72.1	36 (21.8)	0.678 (0.389–1.181)	0.170	0.324	50.0	18 (22.2)	0.794 (0.385–1.633)	0.530	0.530	20.1
***DROSHA/XPO5***																
TT/AA	90 (37.8)	74 (42.8)	1.000 (reference)				59 (35.8)	1.000 (reference)				24 (29.6)	1.000 (reference)			
TT/AC+CC	20 (8.4)	11 (6.4)	0.684 (0.308–1.521)	0.351	0.629	27.8	17 (10.3)	1.261 (0.610–2.607)	0.532	0.946	16.0	7 (8.6)	1.269 (0.478–3.368)	0.632	0.632	11.8
TC+CC/AA	107 (45.0)	73 (42.2)	0.838 (0.545–1.287)	0.419	0.629	22.2	73 (44.2)	1.015 (0.651–1.583)	0.946	0.946	5.9	41 (50.6)	1.438 (0.807–2.561)	0.217	0.471	40.7
TC+CC/AC+CC	21 (8.8)	15 (8.7)	0.904 (0.431–1.894)	0.789	0.789	9.4	16 (9.7)	1.130 (0.542–2.355)	0.745	0.946	9.6	9 (11.1)	1.599 (0.641–3.988)	0.314	0.471	27.5
***RAN/XPO5***																
CC/AA	103 (43.3)	96 (55.5)	1.000 (reference)				81 (49.1)	1.000 (reference)				44 (54.3)	1.000 (reference)			
CC/AC+CC	20 (8.4)	13 (7.5)	0.703 (0.331–1.491)	0.358	0.358	25.6	20 (12.1)	1.233 (0.620–2.453)	0.551	0.551	14.4	9 (11.1)	1.015 (0.426–2.419)	0.973	0.973	4.6
CT+TT/AA	94 (39.5)	51 (29.5)	0.592 (0.381–0.920)	0.020	0.060	79.8	51 (30.9)	0.682 (0.436–1.066)	0.093	0.279	56.4	21 (25.9)	0.522 (0.289–0.943)	0.031	0.093	76.9
CT+TT/AC+CC	21 (8.8)	13 (7.5)	0.687 (0.323–1.462)	0.330	0.358	27.9	13 (7.9)	0.709 (0.326–1.542)	0.385	0.551	22.8	7 (8.6)	0.666 (0.249–1.781)	0.418	0.627	22.9

Note: For AOR, OR was adjusted by age of participants. RPL = recurrent pregnancy loss; PL = number of pregnancy losses; AOR = adjusted odds ratio; CI = confidence interval; HWE = Hardy–Weinberg equilibrium.

a Fisher's exact test;

b FDR-adjusted P value.

To evaluate whether gene-gene interaction models exert synergistic effects on RPL risk, we conducted MDR-based allele combinations of four polymorphisms of miRNA machinery genes (Table 4 and Table S4). We selected two interaction models (*DICER*/*DROSHA*/*RAN*/*XPO5* and *DICER*/*DROSHA*) because their CV values were 10/10. Among the models of four polymorphic loci in *DICER*/*DROSHA*/*RAN*/*XPO5*, four allele combinations (A-T-C-A, A-T-T-C, G-T-C-C, and G-C-T-A) showed synergistic effects on increased RPL risk (OR = 1.510, 95% CI = 1.161–1.965; OR = 20.858, 95% CI = 1.240–350.756; OR = 12.117, 95% CI = 0.697–210.595; and OR = 29.759, 95% CI = 1.794–493.586, respectively), while A-T-C-C, A-C-T-A, and G-T-T-A were associated with reduced RPL risk (OR = 0.366, 95% CI = 0.189–0.709; OR = 0.466, 95% CI = 0.281–0.773; and OR = 0.533, 95% CI = 0.335–0.850, respectively). However, it was difficult to make a conclusion with four allele combinations (*DICER/DROSHA/RAN/XPO5*) of A-T-T-C, G-T-C-C, and G-C-T-A due to the wide range of confidence intervals. Among the models of two loci, the A-T and G-C allele combinations in the *DICER*/*DROSHA* model were associated with increased prevalence of RPL (OR = 1.292, 95% CI = 1.017–1.643 and OR = 1.694, 95% CI = 1.182–2.426, respectively), whereas A-C was associated with reduced RPL risk (OR = 0.615, 95% CI = 0.451–0.839). Table S5 represents the statistical power of the significant genotype. In addition, we conducted power analysis to detect the associations in the case-control study at the 5% significance level. The power varies for an OR by the proportion of exposure (i.e., the target genotype) in the control. A total sample size of 576 (338 RPL cases vs. 238 controls) could have reasonable power to detect an OR of 1.7 when the proportions of exposure in the control were 20% (power = 76.3%), 30% (power = 84.9%), and 40% (power = 87.7%). Additionally, this sample size has reasonable power to detect an OR of 0.55 for the proportions of 20% (power = 73.1%), 30% (power = 85.6%), and 40% (power = 91.3%) in the control. Furthermore, this sample size might have sufficient power to detect an OR of 1.7 or greater and an OR of 0.55 or less. However, the power will be smaller when it is compared with multiple genotypes.

**Table 4:** Allele combination analysis of miRNA machinery genes in RPL patients and control participants using the multifactor dimensionality reduction method.

Allele combination	Controls (n = 238)	RPL patients (n = 338)	OR (95% CI)	*P* a	*P* b	Statistical power (%)
***DICER/DROSHA/RAN/XPO5***						
A-T-C-A	0.2455	0.33	1.510 (1.161–1.965)	0.002	0.010	71.4
A-T-C-C	0.0548	0.0211	0.366 (0.189–0.709)	0.003	0.010	70.0
A-T-T-A	0.0726	0.0636	0.856 (0.539–1.359)	0.552	0.589	11.5
A-T-T-C	0.0001	0.021	20.858 (1.240–350.756)	0.001	0.008	8.6
A-C-C-A	0.1175	0.0874	0.717 (0.487–1.055)	0.110	0.176	31.7
A-C-C-C	0.0057	0.0143	2.367 (0.648–8.651)	0.259	0.345	26.8
A-C-T-A	0.0812	0.0395	0.466 (0.281–0.773)	0.003	0.010	66.6
A-C-T-C	0.0067	0	0.100 (0.005–1.942)	0.070	0.140	16.6
G-T-C-A	0.2176	0.195	0.868 (0.650–1.159)	0.336	0.384	16.4
G-T-C-C	0	0.0114	12.117 (0.697–210.595)	0.024	0.055	
G-T-T-A	0.0893	0.0504	0.533 (0.335–0.850)	0.008	0.021	59.4
G-T-T-C	0.0092	0.0057	0.702 (0.175–2.823)	0.724	0.724	12.3
G-C-C-A	0.086	0.1077	1.284 (0.859–1.920)	0.231	0.336	21.5
G-C-C-C	0.0083	0.0215	2.678 (0.883–8.121)	0.098	0.174	37.0
G-C-T-A	0	0.0302	29.759 (1.794–493.586)	<.0001	0.002	
G-C-T-C	0.0056	0.0011	0.234 (0.024–2.254)	0.313	0.384	9.1
***DICER/DROSHA***						
A-T	0.3761	0.4373	1.292 (1.017–1.643)	0.039	0.052	43.8
A-C	0.208	0.1397	0.615 (0.451–0.839)	0.002	0.008	69.3
G-T	0.313	0.261	0.773 (0.596–1.001)	0.054	0.054	38.9
G-C	0.1029	0.1621	1.694 (1.182–2.426)	0.004	0.008	67.9

Note: ORs and 95% CIs of each allele combinations were calculated with reference to frequencies of all others using Fisher's exact test. *P* value by Fisher's exact test. RPL = recurrent pregnancy loss; AOR = adjusted odds ratio; CI = confidence interval.

a Fisher's exact test;

b FDR-adjusted *P* Value.

## Discussion

We evaluated four SNPs in *DICER*, *DROSHA*, *XPO5*, and *RAN*, which are essential for miRNA biogenesis [26], [27]. Our results comparing differences in genotype frequencies in miRNA machinery genes revealed that *DICER* rs3742330 GG genotype had significant associations with RPL in Korean women when it was combined with the *DROSHA* rs10719 TC+CC genotype. In addition, the ORs of RPL prevalence were higher because patients who possessed the *DICER* rs3742330 GG/*DROSHA* rs10719 TC+CC genotype experienced more pregnancy loss. Defective DICER function due to genetic polymorphism might be intensified by *DROSHA* polymorphisms. These two molecules are considered major regulators of miRNA biogenesis. However, there is no evidence that these molecules interact with each other. It remains unclear how genetic polymorphisms in these genes influence each other. To elucidate the mechanisms by which different alleles of *DICER* or *DROSHA* affect RPL development, additional functional studies should be performed. Although the exact mechanism could not be elucidated in the present study, our finding might be used in the future as a marker to predict RPL development in women with a spontaneous abortion.

Dicer is essential in miRNA biogenesis, and its function has been reported to be involved reproduction. Dicer expression is detected in female reproductive organs, including the ovary, oviduct, and uterus [28]–[30]. Female mice with general hypomorphic mutation of *Dicer* were infertile because of luteal deficiency [30]. The conditional knockout of *Dicer* within ovarian granulosa cells affects ovulation rate by influencing the number of preovulatory follicles that achieve proper development [28], [29]. On the other hand, Dicer is reported to be involved in regulating placental growth by influencing pro-mitogenic signaling molecules through miRNA action [31].

Although there is limited evidence that polymorphisms of *DICER* alter biologic functions depending on alleles, a number of studies have reported that *DICER* polymorphisms, including rs3742330, affect disease development and patient survival in various cancers [31]–[33]. In addition, the functional analysis of the *DICER* rs1057035 SNP, which resides in the 3′UTR of *DICER*, has revealed that targeting of has-miR-574-3p to *DICER* was differentially affected by the type of *DICER* rs1057035 allele and that the *DICER* rs1057035 variant C allele led to reduced expression of Dicer [34]. In the *DICER* gene, the polymorphic site *DICER* rs3742330A>G, found in the 3′ UTR of *DICER*, is considered to be important for mRNA transcript stability. In terms of human reproduction, *DICER* rs12323635, located in promoter region of *DICER*, has been reported to decrease the risk of oligozoospermia [35]. Therefore, Dicer is likely to be a crucial molecule for pregnancy.

There are several potential mechanisms that explain why *DICER* polymorphisms are associated with miscarriage. *DICER* polymorphism may affect decidualization of the endometrium and induce implantation defects in embryos, resulting in recurrent miscarriage. Decidualization of endometrial stroma is one of the crucial steps for trophoblast invasion and placenta development [36]. A recent study using human embryonic stem cells demonstrated that DICER expression increased during *in vitro* decidualization. DICER inhibition affected the expression of decidual markers and transcription factors—such as PRL and HOXA10—which play a role during decidualization [37]. Second, *DICER* polymorphisms may have an influence on placental development by affecting miRNA expression involving cytotrophoblast proliferation. Cytotrophoblasts are trophoblastic stem cells that play multiple roles [6]. Recent research has demonstrated enhanced proliferation of cytotrophoblasts without exogenous growth factors following Dicer knock-down in first-trimester placenta. Although the miRNAs affecting cell proliferation were not identified, the authors suggested that Dicer-dependent miRNA affected the proliferation of cytotrophoblasts [5]. Dicer polymorphisms induce aberrant expression of miRNAs regulating cytotrophoblast proliferation, possibly resulting in abnormal placental development and abortion. Finally, the miRNA expression affected by the *DICER* polymorphism may induce chromosome mis-segregation, leading to aneuploidy, which is the most common cause of miscarriage. The centrosome is known to be a source of aneuploidy. The extra centrosome forms a multipolar spindle during mitosis in a mother cell, resulting in aneuploid daughter cells. This pathogenesis has been shown in cancer development [38]. miR-210 overexpression is reported to induce centrosome amplification in renal cancer [39]. Therefore, aberrant miRNA expression may cause chromosomal aneuploidy in cancer. A similar mechanism exists by which abnormal miRNA expression may cause aneuploid embryos and recurrent miscarriage during early development. A significant association between the *RAN* rs14035 genotype and RPL prevalence was observed when it was combined with the *XPO5* rs11077 AA genotype. Ran, which is a member of the Ras superfamily of GTPases, is essential for the translocation of pre-miRNAs from the nucleus to the cytoplasm through the nuclear pore complex [40]. Xpo5 mediates nuclear export of pre-miRNA in a RAN GTP-dependent manner by binding to pre-miRNA and RAN GTPase in the nucleus [40]. Although several reports have shown the association of these molecules with disease development in various cancers, no report has elucidated the role of these molecules in human reproduction [41], [42].

Our previous study revealed that the *XPO5* rs2257082 allele was more frequently observed in premature ovarian insufficiency (POI) patients than in control subjects [20]. However, we could not identify any association between *RAN* polymorphism and POI development. Additionally, *RAN* rs14035, but not *XPO5* rs11077, exerted an effect on RPL development. Regarding both molecules, few reports have evaluated the clinical significance of SNPs not only in cancer but also in pregnancy-related disease. In addition, no study has elucidated the functions of those SNPs in miRNA biogenesis. Therefore, additional functional studies are required to elucidate how *RAN* rs14035 affects disease promotion and prevention depending on its genotype.

We obtained contrasting results regarding RPL development in the combination analysis for the *DROSHA* rs10719 TC+CC genotype. When it was combined with *DICER* rs3724330 GG genotype, RPL development was more frequently observed in RPL group. When the *DROSHA* rs10719 TC+CC genotype was combined with the *RAN* rs14035 CT+TT genotype, RPL development was rare in the RPL group. One potential explanation for this observation is that idiopathic RPL has various unknown etiologies. Many reports in the literature have demonstrated that several causes are associated with recurrent miscarriage, such as angiogenesis, immunologic factors, and miRNAs [15], [18], [43]. This may explain why the same polymorphism affects disease development differently. The result of our combination analysis of polymorphisms according to the number of pregnancy losses supports this explanation (Table 3). The AORs increased as the number of previous pregnancy losses increased in patients who have the *DROSHA* rs10719 TC+CC genotype combined with the *DICER* rs3742330 GG genotype (1.596 vs. 2.460 vs. 4.084). Conversely, no such tendency was noted with the *DROSHA* TC+CC/*RAN* CT+TT genotype. Instead, the effect of polymorphisms was attenuated as the number of miscarriages increased. This suggests that genetic polymorphisms with respect to miRNA machinery genes were associated with disease development not through a common pathway but through various pathogeneses.

There are several potential limitations in this study. First, this case-control study only shows an association between SNPs in miRNA machinery genes and RPL development, not a causal relationship. Therefore, it is difficult to conclude that miRNA machinery genes affect recurrent miscarriage. Second, the manner in which polymorphisms in miRNA biogenesis pathway genes affect RPL development is still unclear, and a functional study of those SNPs to elucidate the pathogenesis related to RPL was not conducted. Third, the lack of information regarding placental pathology, immunologic profiles, and miRNA expression could have contributed to the investigation of potential roles of miRNA machinery genes during the peri-implantation and early pregnancy periods. Lastly, the sample size of the control group is relatively small, compared with that of the study group. This is because we included all eligible cases during the study period to avoid selection bias. This difference in sample size will increase type 2 errors in the genetic association study. To reduce the bias, we compared the *RAN* rs14035 and *XPO5* rs11077 genotype frequencies in the control group with those from other studies that were performed in Korea. The genotype frequencies of *RAN* rs14035 and *XPO5* rs11077 in the control group of our study showed similar patterns to the results in the control groups of previous reports regarding lung cancer and breast cancer in Korea [26], [27].

Recent groundbreaking discoveries that small, non-coding RNAs can regulate gene expression transcriptionally or post-transcriptionally and that these RNAs are involved in placental development through trophoblast differentiation, proliferation, and angiogenesis provide us with insight into the pathogenesis of idiopathic RPL [4], [7], [44]. To the best of our knowledge, this is the first report to investigate the association between polymorphisms in miRNA biogenesis machinery genes and the prevalence of RPL in a Korean population and suggests that genetic polymorphisms in miRNA biogenesis machinery genes show a relationship with the risk of RPL. The present study may contribute to assessments of individual risk of RPL. However, further epidemiologic studies with larger subject numbers should be performed to confirm and expand our results. In addition, the results of our study warrant further functional studies to elucidate the mechanisms by which polymorphisms of miRNA machinery genes affect RPL development.

## Supporting Information

Table S1 **Participant characteristics.**
(DOCX)

Table S2 **Single-nucleotide polymorphisms of miRNA machinery genes.**
(DOCX)

Table S3 **Comparison of genotype frequencies of polymorphisms in miRNA machinery genes between RPL and control subjects according to the number of previous pregnancy losses.**
(DOCX)

Table S4 **Allele combinations of polymorphisms in miRNA machinery genes between RPL and control subjects according to the number of previous pregnancy losses.**
(DOCX)

Table S5 **Power calculation to detect an association between a single gene and recurrent pregnancy loss (RPL) with a two-tailed significance level of 0.05 under a case-control design.**
(DOCX)

## References

[1] Wilcox AJ, Weinberg CR, O'Connor JF, Baird DD, Schlatterer JP, et al (1988) Incidence of early loss of pregnancy. N Engl J Med 319: 189–194. 3393170 10.1056/NEJM198807283190401

[2] Stirrat GM (1990) Recurrent miscarriage. II: Clinical associations, causes, and management. Lancet 336: 728–733. 1975901 10.1016/0140-6736(90)92215-4

[3] Practice Committee of the American Society for Reproductive Medicine (2012) Evaluation and treatment of recurrent pregnancy loss: a committee opinion. Fertil Steril 98: 1103–1111. 22835448 10.1016/j.fertnstert.2012.06.048

[4] Donker RB, Mouillet JF, Nelson DM, Sadovsky Y (2007) The expression of Argonaute2 and related microRNA biogenesis proteins in normal and hypoxic trophoblasts. Mol Hum Reprod 13: 273–279. 17327266 10.1093/molehr/gam006

[5] Forbes K, Farrokhnia F, Aplin JD, Westwood M (2012) Dicer-dependent miRNAs provide an endogenous restraint on cytotrophoblast proliferation. Placenta 33: 581–585. 22516645 10.1016/j.placenta.2012.03.006

[6] Fu G, Brkic J, Hayder H, Peng C (2013) MicroRNAs in Human Placental Development and Pregnancy Complications. Int J Mol Sci 14: 5519–5544. 23528856 10.3390/ijms14035519 PMC3634453

[7] Chakrabarty A, Tranguch S, Daikoku T, Jensen K, Furneaux H, et al (2007) MicroRNA regulation of cyclooxygenase-2 during embryo implantation. Proc Natl Acad Sci U S A 104: 15144–15149. 17848513 10.1073/pnas.0705917104 PMC1986627

[8] Zhu XM, Han T, Wang XH, Li YH, Yang HG, et al (2010) Overexpression of miR-152 leads to reduced expression of human leukocyte antigen-G and increased natural killer cell mediated cytolysis in JEG-3 cells. Am J Obstet Gynecol 202: e591–597, 592, e591-597. 10.1016/j.ajog.2010.03.002 20430358

[9] Enquobahrie DA, Abetew DF, Sorensen TK, Willoughby D, Chidambaram K, et al (2011) Placental microRNA expression in pregnancies complicated by preeclampsia. Am J Obstet Gynecol 204: e112–121, 178, e112-121. 10.1016/j.ajog.2010.09.004 PMC3040986 21093846

[10] Mayor-Lynn K, Toloubeydokhti T, Cruz AC, Chegini N (2011) Expression profile of microRNAs and mRNAs in human placentas from pregnancies complicated by preeclampsia and preterm labor. Reprod Sci 18: 46–56. 21079238 10.1177/1933719110374115 PMC3343068

[11] Mouillet JF, Chu T, Hubel CA, Nelson DM, Parks WT, et al (2010) The levels of hypoxia-regulated microRNAs in plasma of pregnant women with fetal growth restriction. Placenta 31: 781–784. 20667590 10.1016/j.placenta.2010.07.001 PMC3204658

[12] Ohlsson Teague EM, Van der Hoek KH, Van der Hoek MB, Perry N, Wagaarachchi P, et al (2009) MicroRNA-regulated pathways associated with endometriosis. Mol Endocrinol 23: 265–275. 19074548 10.1210/me.2008-0387 PMC5419313

[13] Pan Q, Luo X, Toloubeydokhti T, Chegini N (2007) The expression profile of micro-RNA in endometrium and endometriosis and the influence of ovarian steroids on their expression. Mol Hum Reprod 13: 797–806. 17766684 10.1093/molehr/gam063

[14] Choi YS, Kwon H, Kim JH, Shin JE, Choi Y, et al (2011) Haplotype-based association of ACE I/D, AT1R 1166A>C, and AGT M235T polymorphisms in renin-angiotensin-aldosterone system genes in Korean women with idiopathic recurrent spontaneous abortions. Eur J Obstet Gynecol Reprod Biol 158: 225–228. 21636204 10.1016/j.ejogrb.2011.04.028

[15] Jeon YJ, Choi YS, Rah H, Kim SY, Choi DH, et al (2012) Association study of microRNA polymorphisms with risk of idiopathic recurrent spontaneous abortion in Korean women. Gene 494: 168–173. 22222140 10.1016/j.gene.2011.12.026

[16] Miyamura H, Nishizawa H, Ota S, Suzuki M, Inagaki A, et al (2011) Polymorphisms in the annexin A5 gene promoter in Japanese women with recurrent pregnancy loss. Mol Hum Reprod 17: 447–452. 21289001 10.1093/molehr/gar008

[17] Kim JH, Jeon YJ, Lee BE, Kang H, Shin JE, et al (2013) Association of methionine synthase and thymidylate synthase genetic polymorphisms with idiopathic recurrent pregnancy loss. Fertil Steril 99: 1674–1680. 23415967 10.1016/j.fertnstert.2013.01.108

[18] Hu Y, Liu CM, Qi L, He TZ, Shi-Guo L, et al (2011) Two common SNPs in pri-miR-125a alter the mature miRNA expression and associate with recurrent pregnancy loss in a Han-Chinese population. RNA Biol 8: 861–872. 21788734 10.4161/rna.8.5.16034

[19] Lei B, Gao S, Luo LF, Xia XY, Jiang SW, et al (2011) A SNP in the miR-27a gene is associated with litter size in pigs. Mol Biol Rep 38: 3725–3729. 21104015 10.1007/s11033-010-0487-2

[20] Rah H, Jeon YJ, Lee BE, Kim JO, Shim SH, et al (2013) Association of polymorphisms in microRNA machinery genes (DROSHA, DICER1, RAN, and XPO5) with risk of idiopathic primary ovarian insufficiency in Korean women. Menopause 20: 1067–1073. 23549446 10.1097/GME.0b013e3182883907

[21] Barch MJ, Knutsen T, Spurbeck JL, Association of Genetic Technologists (1997) The AGT cytogenetics laboratory manual. Philadelphia: Lippincott-Raven Publishers. 325–374 p.

[22] Carp H, Toder V, Aviram A, Daniely M, Mashiach S, et al (2001) Karyotype of the abortus in recurrent miscarriage. Fertil Steril 75: 678–682. 11287018 10.1016/s0015-0282(00)01801-x

[23] Hahn LW, Ritchie MD, Moore JH (2003) Multifactor dimensionality reduction software for detecting gene-gene and gene-environment interactions. Bioinformatics 19: 376–382. 12584123 10.1093/bioinformatics/btf869

[24] Lee SY, Chung Y, Elston RC, Kim Y, Park T (2007) Log-linear model-based multifactor dimensionality reduction method to detect gene gene interactions. Bioinformatics 23: 2589–2595. 17872915 10.1093/bioinformatics/btm396

[25] Ritchie MD, Hahn LW, Roodi N, Bailey LR, Dupont WD, et al (2001) Multifactor-dimensionality reduction reveals high-order interactions among estrogen-metabolism genes in sporadic breast cancer. Am J Hum Genet 69: 138–147. 11404819 10.1086/321276 PMC1226028

[26] Kim JS, Choi YY, Jin G, Kang HG, Choi JE, et al (2010) Association of a common AGO1 variant with lung cancer risk: a two-stage case-control study. Mol Carcinog 49: 913–921. 20721975 10.1002/mc.20672

[27] Sung H, Lee KM, Choi JY, Han S, Lee JY, et al (2011) Common genetic polymorphisms of microRNA biogenesis pathway genes and risk of breast cancer: a case-control study in Korea. Breast Cancer Res Treat 130: 939–951. 21766210 10.1007/s10549-011-1656-2

[28] Hong X, Luense LJ, McGinnis LK, Nothnick WB, Christenson LK (2008) Dicer1 is essential for female fertility and normal development of the female reproductive system. Endocrinology 149: 6207–6212. 18703631 10.1210/en.2008-0294 PMC2613048

[29] Nagaraja AK, Andreu-Vieyra C, Franco HL, Ma L, Chen R, et al (2008) Deletion of Dicer in somatic cells of the female reproductive tract causes sterility. Mol Endocrinol 22: 2336–2352. 18687735 10.1210/me.2008-0142 PMC2582529

[30] Otsuka M, Zheng M, Hayashi M, Lee JD, Yoshino O, et al (2008) Impaired microRNA processing causes corpus luteum insufficiency and infertility in mice. J Clin Invest 118: 1944–1954. 18398510 10.1172/JCI33680 PMC2289794

[31] Clague J, Lippman SM, Yang H, Hildebrandt MA, Ye Y, et al (2010) Genetic variation in MicroRNA genes and risk of oral premalignant lesions. Mol Carcinog 49: 183–189. 19851984 10.1002/mc.20588 PMC3640857

[32] Li X, Tian X, Zhang B, Zhang Y, Chen J (2012) Variation in dicer gene is associated with increased survival in T-cell lymphoma. PLoS One 7: e51640. 23251602 10.1371/journal.pone.0051640 PMC3518478

[33] Lin J, Horikawa Y, Tamboli P, Clague J, Wood CG, et al (2010) Genetic variations in microRNA-related genes are associated with survival and recurrence in patients with renal cell carcinoma. Carcinogenesis 31: 1805–1812. 20732906 10.1093/carcin/bgq168 PMC2981457

[34] Ma H, Yuan H, Yuan Z, Yu C, Wang R, et al (2012) Genetic variations in key microRNA processing genes and risk of head and neck cancer: a case-control study in Chinese population. PLoS One 7: e47544. 23071822 10.1371/journal.pone.0047544 PMC3469541

[35] Qin Y, Xia Y, Wu W, Han X, Lu C, et al (2012) Genetic variants in microRNA biogenesis pathway genes are associated with semen quality in a Han-Chinese population. Reprod Biomed Online 24: 454–461. 22381205 10.1016/j.rbmo.2012.01.006

[36] Gellersen B, Brosens IA, Brosens JJ (2007) Decidualization of the human endometrium: mechanisms, functions, and clinical perspectives. Semin Reprod Med 25: 445–453. 17960529 10.1055/s-2007-991042

[37] Estella C, Herrer I, Moreno-Moya JM, Quinonero A, Martinez S, et al (2012) miRNA signature and Dicer requirement during human endometrial stromal decidualization in vitro. PLoS One 7: e41080. 22911744 10.1371/journal.pone.0041080 PMC3401238

[38] Holland AJ, Cleveland DW (2009) Boveri revisited: chromosomal instability, aneuploidy and tumorigenesis. Nat Rev Mol Cell Biol 10: 478–487. 19546858 10.1038/nrm2718 PMC3154738

[39] Nakada C, Tsukamoto Y, Matsuura K, Nguyen TL, Hijiya N, et al (2011) Overexpression of miR-210, a downstream target of HIF1alpha, causes centrosome amplification in renal carcinoma cells. J Pathol 224: 280–288. 21465485 10.1002/path.2860

[40] Lund E, Guttinger S, Calado A, Dahlberg JE, Kutay U (2004) Nuclear export of microRNA precursors. Science 303: 95–98. 14631048 10.1126/science.1090599

[41] Lin M, Gu J, Eng C, Ellis LM, Hildebrandt MA, et al (2012) Genetic polymorphisms in MicroRNA-related genes as predictors of clinical outcomes in colorectal adenocarcinoma patients. Clin Cancer Res 18: 3982–3991. 22661538 10.1158/1078-0432.CCR-11-2951 PMC4141857

[42] Ye Y, Wang KK, Gu J, Yang H, Lin J, et al (2008) Genetic variations in microRNA-related genes are novel susceptibility loci for esophageal cancer risk. Cancer Prev Res (Phila) 1: 460–469. 19138993 10.1158/1940-6207.CAPR-08-0135 PMC2768267

[43] Lee SK, Kim JY, Hur SE, Kim CJ, Na BJ, et al (2011) An imbalance in interleukin-17-producing T and Foxp3(+) regulatory T cells in women with idiopathic recurrent pregnancy loss. Hum Reprod 26: 2964–2971. 21926059 10.1093/humrep/der301

[44] Dai XY, Zeng XX, Peng F, Han YY, Lin HJ, et al (2012) A novel anticancer agent, SKLB70359, inhibits human hepatic carcinoma cells proliferation via G0/G1 cell cycle arrest and apoptosis induction. Cell Physiol Biochem 29: 281–290. 22415097 10.1159/000337609

