# Correction: Genetic Variants in MicroRNA Machinery Genes Are Associate with Idiopathic Recurrent Pregnancy Loss Risk

**DOI:** 10.1371/journal.pone.0105743

**Published:** 2014-08-08

**Authors:** The PLOS ONE Staff

The word “Associate” is incorrect in the article title. The correct title is: Genetic Variants in MicroRNA Machinery Genes Are Associated with Idiopathic Recurrent Pregnancy Loss Risk. The correct citation is: Jung YW, Jeon YJ, Rah H, Kim JH, Shin JE, et al. (2014) Genetic Variants in MicroRNA Machinery Genes Are Associated with Idiopathic Recurrent Pregnancy Loss Risk. PLoS ONE 9(4): e95803. doi:10.1371/journal.pone.0095803
