# Supplementary material for: Genetic Variants in MicroRNA Machinery Genes Are Associate with Idiopathic Recurrent Pregnancy Loss Risk
Source: PLoS One. 2014 Apr 25;9(4):e95803. doi: 10.1371/journal.pone.0095803 (PMC4000197; doi:10.1371/journal.pone.0095803)
Supplement: Table S1 — Participant characteristics. (DOCX) [file pone.0095803.s001.docx]

| **Table S1**  **Participant characteristics.** | | |
| --- | --- | --- |
| **Characteristics** | **Controls (n=238)** | **RPL patients (n=338)** |
| Age (years) | 33.38 ± 5.79 | 32.81 ± 4.33 |
| BMI (kg/m^2^) | 21.68 ± 3.10 | 21.40 ± 4.87 |
| Previous pregnancy losses | NA | 3.04 ± 1.61 |
| RPL < 14 weeks | NA | 99.70% |
| Live births | 1.71 ± 0.72 | NA |
| Average gestational age (weeks) | 39.28 ± 1.67 | 7.32 ± 1.85 |
| Note: RPL = recurrent pregnancy loss; BMI = body mass index; NA = not applicable. | | |
