# Supplementary material for: Genetic Variants in MicroRNA Machinery Genes Are Associate with Idiopathic Recurrent Pregnancy Loss Risk
Source: PLoS One. 2014 Apr 25;9(4):e95803. doi: 10.1371/journal.pone.0095803 (PMC4000197; doi:10.1371/journal.pone.0095803)
Supplement: Table S2 — Single-nucleotide polymorphisms of miRNA machinery genes. (DOCX) [file pone.0095803.s002.docx]

**Table S2**

**Single-nucleotide polymorphisms of miRNA machinery genes.**

| **Gene** | **Polymorphism** | **Position** | **Nucleotide change** | **MAF^a^** | **PCR forward primer** | **PCR reverse primer** |
| --- | --- | --- | --- | --- | --- | --- |
| *DROSHA* | rs10719 | 3' UTR | C>T | 0.768 | CTA GTT TTC CTG CAG ACA ATG CA | GTA ATG CAC ATT CAC CAA AGT CA |
| *DICER1* | rs3742330 | 3' UTR | A>G | 0.419 | GGT CTC AGT TTG GTG GCT TC | CCT GCC TTG ACA ACA TGA AA |
| *RAN* | rs14035 | 3' UTR | C>T | 0.308 | GAA GCA CTT GCT CAA AAT CTG TGA C | TGC CAT CCA CTG ATG TTC CAT C |
| *XPO5* | rs11077 | 3' UTR | A>C | 0.064 | TGC TTT GGG CAA GAA TCT GGT CAC | TAA AGG GGA TGT TAG CAC TAA AGA AT |

MAF = minor allele frequency; mismatched sequence is underlined.

^a^MAF of Japanese subjects in Tokyo, Japan in Hapmap database.
