# Supplementary material for: Genetic Variants in MicroRNA Machinery Genes Are Associate with Idiopathic Recurrent Pregnancy Loss Risk
Source: PLoS One. 2014 Apr 25;9(4):e95803. doi: 10.1371/journal.pone.0095803 (PMC4000197; doi:10.1371/journal.pone.0095803)
Supplement: Table S3 — Comparison of genotype frequencies of polymorphisms in miRNA machinery genes between RPL and control subjects according to the number of previous pregnancy losses. (DOCX) [file pone.0095803.s003.docx]

| **Table S3**  **Comparison of genotype frequencies of polymorphisms in miRNA machinery genes between RPL and control subjects according to the number of previous pregnancy losses.** | | | | | | | | | | | | | | |
| --- | --- | --- | --- | --- | --- | --- | --- | --- | --- | --- | --- | --- | --- | --- |
| **Genotypes** | **Controls** | **PL=2** | **AOR (95% CI)** | ***P^a^*** | ***P^b^*** | | **PL≥3** | **AOR (95% CI)** | ***P^a^*** | ***P^b^*** | **PL≥4** | **AOR (95% CI)** | ***P^a^*** | ***P^b^*** |
|  | **(n=238)** | **(n=173)** |  |  |  |  | **(n=165)** |  |  |  | **(n=81)** |  |  |  |
| ***DICER* rs3742330** | | | | | | | | | | | | | |  |
| AA | 75 (31.5) | 61 (35.3) | 1.000 (reference) |  |  | 58 (35.2) | | 1.000 (reference) |  |  | 27 (33.3) | 1.000 (reference) |  |  |
| AG | 123 (51.7) | 83 (48.0) | 0.770 (0.496 - 1.195) | 0.244 | 0.488 | 69 (41.8) | | 0.705 (0.449 - 1.107) | 0.129 | 0.258 | 32 (39.6) | 0.693 (0.386 - 1.246) | 0.221 | 0.295 |
| GG | 35 (14.7) | 29 (16.8) | 0.993 (0.545 - 1.808) | 0.980 | 0.980 | 38 (23.0) | | 1.397 (0.787 - 2.478) | 0.254 | 0.469 | 22 (27.2) | 1.734 (0.867 - 3.469) | 0.120 | 0.480 |
| Dominant (AA vs. AG+GG) | | | 0.817 (0.538 - 1.241) | 0.344 | 0.629 |  | | 0.855 (0.562 - 1.302) | 0.466 | 0.621 |  | 0.921 (0.538 - 1.576) | 0.763 | 0.763 |
| Recessive (AA+AG vs. GG) | | | 1.154 (0.673 - 1.976) | 0.603 | 0.832 |  | | 1.718 (1.032 - 2.861) | 0.038 | 0.152 |  | 2.170 (1.181 - 3.986) | 0.013 | 0.052 |
| HWE *P* | 0.18 | 0.932 |  |  |  | 0.052 | |  |  |  | 0.063 |  |  |  |
| ***DROSHA* rs10719** | | | | | | | | | | | | | |  |
| TT | 110 (46.2) | 85 (49.1) | 1.000 (reference) |  |  | 76 (46.1) | | 1.000 (reference) |  |  | 31 (38.3) | 1.000 (reference) |  |  |
| TC | 108 (45.4) | 70 (40.5) | 0.855 (0.565 - 1.294) | 0.457 | 0.541 | 80 (48.5) | | 1.052 (0.697 - 1.586) | 0.810 | 0.810 | 44 (54.3) | 1.445 (0.850 - 2.457) | 0.174 | 0.295 |
| CC | 20 (8.4) | 18 (10.4) | 1.100 (0.541 - 2.234) | 0.793 | 0.980 | 9 (5.5) | | 0.670 (0.288 - 1.557) | 0.352 | 0.469 | 6 (7.4) | 1.128 (0.413 - 3.077) | 0.815 | 0.952 |
| Dominant (TT vs. TC+CC) | | | 0.895 (0.604 - 1.328) | 0.583 | 0.629 |  | | 0.988 (0.664 - 1.471) | 0.953 | 0.953 |  | 1.387 (0.828 - 2.322) | 0.214 | 0.428 |
| Recessive (TT+TC vs. CC) | | | 1.188 (0.604 - 2.335) | 0.618 | 0.832 |  | | 0.618 (0.274 - 1.394) | 0.246 | 0.488 |  | 0.873 (0.337 - 2.257) | 0.779 | 0.779 |
| HWE *P* | 0.363 | 0.528 |  |  |  | 0.763 | |  |  |  | 0.071 |  |  |  |
| ***RAN* rs14035** | | | | | | | | | | | | | |  |
| CC | 123 (51.7) | 109 (63.0) | 1.000 (reference) |  |  | 101 (61.2) | | 1.000 (reference) |  |  | 53 (65.4) | 1.000 (reference) |  |  |
| CT | 104 (43.7) | 56 (32.4) | 0.624 (0.412 - 0.947) | 0.027 | 0.108 | 57 (34.5) | | 0.661 (0.436 - 1.003) | 0.052 | 0.208 | 23 (28.4) | 0.502 (0.287 - 0.879) | 0.016 | 0.064 |
| TT | 11 (4.6) | 8 (4.6) | 0.846 (0.325 - 2.205) | 0.733 | 0.980 | 7 (4.2) | | 0.763 (0.285 - 2.044) | 0.591 | 0.591 | 5 (6.2) | 1.035 (0.341 - 3.143) | 0.952 | 0.952 |
| Dominant (CC vs. CT+TT) | | | 0.648 (0.434 - 0.969) | 0.035 | 0.140 |  | | 0.674 (0.450 - 1.009) | 0.055 | 0.220 |  | 0.561 (0.332 - 0.951) | 0.032 | 0.128 |
| Recessive (CC+CT vs. TT) | | | 1.082 (0.422 - 2.775) | 0.869 | 0.869 |  | | 0.908 (0.344 - 2.395) | 0.846 | 0.846 |  | 1.357 (0.457 - 4.032) | 0.583 | 0.777 |
| HWE *P* | 0.059 | 0.814 |  |  |  | 0.769 | |  |  |  | 0.262 |  |  |  |
| ***XPO5* rs11077** | | | | | | | | | | | | | |  |
| AA | 197 (82.8) | 147 (85.0) | 1.000 (reference) |  |  | 132 (80.0) | | 1.000 (reference) |  |  | 65 (80.2) | 1.000 (reference) |  |  |
| AC | 39 (16.4) | 23 (13.3) | 0.841 (0.484 - 1.464) | 0.541 | 0.541 | 30 (18.2) | | 1.149 (0.678 - 1.948) | 0.605 | 0.807 | 14 (17.3) | 1.081 (0.550 - 2.126) | 0.821 | 0.821 |
| CC | 2 (0.8) | 3 (1.7) | 1.546 (0.206 - 11.608) | 0.672 | 0.980 | 3 (1.8) | | 2.450 (0.392 - 15.311) | 0.338 | 0.469 | 2 (2.5) | 3.309 (0.432 - 25.349) | 0.249 | 0.498 |
| Dominant (AA vs. AC+CC) | | | 0.876 (0.511 - 1.501) | 0.629 | 0.629 |  | | 1.216 (0.728 - 2.031) | 0.455 | 0.621 |  | 1.184 (0.619 - 2.266) | 0.609 | 0.763 |
| Recessive (AA+AC vs. CC) | | | 1.666 (0.217 - 12.780) | 0.624 | 0.832 |  | | 2.312 (0.376 - 14.235) | 0.366 | 0.488 |  | 3.072 (0.411 - 22.991) | 0.275 | 0.550 |
| HWE *P* | 0.964 | 0.077 |  |  |  | 0.406 | |  |  |  | 0.261 |  |  |  |

Note: AORs and 95% CIs of each genotype were calculated with reference to frequencies of all others. For AOR, OR was adjusted by age of participants. RPL = recurrent pregnancy loss; PL = number of pregnancy losses; AOR = adjusted odds ratio; CI = confidence interval; HWE = Hardy–Weinberg equilibrium. ^a^Fisher’s exact test; ^b^FDR-adjusted *P* value.
