# Supplementary material for: Genetic Variants in MicroRNA Machinery Genes Are Associate with Idiopathic Recurrent Pregnancy Loss Risk
Source: PLoS One. 2014 Apr 25;9(4):e95803. doi: 10.1371/journal.pone.0095803 (PMC4000197; doi:10.1371/journal.pone.0095803)
Supplement: Table S4 — Allele combinations of polymorphisms in miRNA machinery genes between RPL and control subjects according to the number of previous pregnancy losses. (DOCX) [file pone.0095803.s004.docx]

| **Table S4**  **Allele combinations of polymorphisms in miRNA machinery genes between RPL and control subjects according to the number of previous pregnancy losses.** | | | | | | | | | | | | | | |
| --- | --- | --- | --- | --- | --- | --- | --- | --- | --- | --- | --- | --- | --- | --- |
| **Allele combination** | **Controls**  **(n=238)** | | **PL=2**  **(n=173)** | **OR (95% CI)** | ***P^a^*** | ***P^b^*** | **PL≥3**  **(n=165)** | **OR (95% CI)** | ***P^a^*** | ***P^b^*** | **PL≥4**  **(n=81)** | **OR (95% CI)** | ***P^a^*** | ***P^b^*** |
| ***DICER/DROSHA/RAN/XPO5*** | | | |  |  |  |  |  |  |  |  |  |  |  |
| A-T-C-A | 0.2455 | 0.3296 | | 1.508 (1.110 - 2.048) | 0.010 | 0.038 | 0.3304 | 1.513 (1.110 - 2.064) | 0.011 | 0.044 | 0.3113 | 1.370 (0.924 - 2.030) | 0.122 | 0.242 |
| A-T-C-C | 0.0548 | 0.0217 | | 0.410 (0.183 - 0.916) | 0.032 | 0.080 | 0.0265 | 0.485 (0.224 - 1.050) | 0.078 | 0.178 | 0.0083 | 0.108 (0.014 - 0.799) | 0.006 | 0.032 |
| A-T-T-A | 0.0726 | 0.0798 | | 1.109 (0.661 - 1.862) | 0.693 | 0.743 | 0.0330 | 0.434 (0.217 - 0.869) | 0.020 | 0.064 | 0.0369 | 0.485 (0.200 - 1.175) | 0.136 | 0.242 |
| A-T-T-C | 0.0001 | 0.0174 | | 18.192 (1.021 - 324.250) | 0.005 | 0.025 | 0.0250 | 25.118 (1.444 - 437.008) | 0.001 | 0.008 | 0.0209 | 20.912 (1.074 - 407.351) | 0.016 | 0.064 |
| A-C-C-A | 0.1175 | 0.0884 | | 0.738 (0.465 - 1.172) | 0.208 | 0.365 | 0.0914 | 0.750 (0.470 - 1.197) | 0.247 | 0.368 | 0.0967 | 0.822 (0.457 - 1.478) | 0.568 | 0.657 |
| A-C-C-C | 0.0057 | 0.0138 | | 2.312 (0.549 - 9.743) | 0.292 | 0.365 | 0.0120 | 1.935 (0.430 - 8.704) | 0.453 | 0.558 | 0.0179 | 2.975 (0.594 - 14.894) | 0.175 | 0.280 |
| A-C-T-A | 0.0812 | 0.0413 | | 0.473 (0.252 - 0.885) | 0.021 | 0.063 | 0.0422 | 0.496 (0.265 - 0.930) | 0.030 | 0.080 | 0.0389 | 0.431 (0.179 - 1.038) | 0.074 | 0.197 |
| A-C-T-C | 0.0067 | 0.0005 | | 0.195 (0.010 - 3.794) | 0.268 | 0.365 | 0.0000 | 0.205 (0.011 - 3.978) | 0.274 | 0.368 | 0.0000 | 0.416 (0.021 - 8.108) | 0.575 | 0.657 |
| G-T-C-A | 0.2176 | 0.2008 | | 0.891 (0.633 - 1.254) | 0.545 | 0.629 | 0.1739 | 0.747 (0.522 - 1.069) | 0.128 | 0.256 | 0.1956 | 0.880 (0.565 - 1.372) | 0.657 | 0.657 |
| G-T-C-C | 0.0000 | 0.0000 | | NA | NA | NA | 0.0250 | 25.118 (1.444 - 437.008) | 0.001 | 0.008 | 0.0290 | 33.279 (1.829 - 605.657) | 0.001 | 0.008 |
| G-T-T-A | 0.0893 | 0.0398 | | 0.425 (0.228 - 0.789) | 0.005 | 0.025 | 0.0892 | 0.970 (0.592 - 1.589) | 1.000 | 1.000 | 0.0393 | 0.387 (0.162 - 0.928) | 0.027 | 0.086 |
| G-T-T-C | 0.0092 | 0.0046 | | 0.686 (0.125 - 3.768) | 1.000 | 1.000 | 0.0000 | 0.159 (0.009 - 2.963) | 0.149 | 0.265 | 0.0131 | 1.475 (0.268 - 8.133) | 0.647 | 0.657 |
| G-C-C-A | 0.0860 | 0.1118 | | 1.348 (0.849 - 2.140) | 0.233 | 0.365 | 0.1097 | 1.299 (0.811 - 2.082) | 0.276 | 0.368 | 0.1155 | 1.410 (0.792 - 2.508) | 0.275 | 0.400 |
| G-C-C-C | 0.0083 | 0.0258 | | 3.151 (0.962 - 10.321) | 0.052 | 0.111 | 0.0159 | 1.815 (0.484 - 6.814) | 0.499 | 0.570 | 0.0220 | 2.987 (0.738 - 12.089) | 0.118 | 0.242 |
| G-C-T-A | 0.0000 | 0.0248 | | 26.825 (1.555 - 462.792) | 0.000 | 0.000 | 0.0211 | 22.094 (1.257 - 388.471) | 0.002 | 0.011 | 0.0546 | 58.980 (3.411 - 1019.979) | <.0001 | 0.002 |
| G-C-T-C | 0.0056 | 0.0000 | | 0.195 (0.010 - 3.794) | 0.268 | 0.365 | 0.0049 | 0.961 (0.160 - 5.788) | 1.000 | 1.000 | 0.0000 | 0.416 (0.021 - 8.108) | 0.575 | 0.657 |
| ***DICER/DROSHA*** | | | |  |  |  |  |  |  |  |  |  |  |  |
| A-T | 0.3761 | 0.4503 | | 1.362 (1.028 - 1.805) | 0.037 | 0.037 | 0.4232 | 1.223 (0.918 - 1.628) | 0.187 | 0.249 | 0.3755 | 1.002 (0.694 - 1.448) | 1.000 | 1.000 |
| A-C | 0.2080 | 0.1422 | | 0.628 (0.432 - 0.914) | 0.017 | 0.034 | 0.1374 | 0.601 (0.409 - 0.883) | 0.009 | 0.036 | 0.1554 | 0.695 (0.430 - 1.123) | 0.167 | 0.334 |
| G-T | 0.3130 | 0.2434 | | 0.704 (0.515 - 0.962) | 0.028 | 0.037 | 0.2798 | 0.848 (0.623 - 1.155) | 0.310 | 0.310 | 0.2788 | 0.844 (0.569 - 1.253) | 0.430 | 0.573 |
| G-C | 0.1029 | 0.1641 | | 1.719 (1.141 - 2.590) | 0.011 | 0.034 | 0.1596 | 1.667 (1.099 - 2.530) | 0.018 | 0.036 | 0.1903 | 2.062 (1.262 - 3.368) | 0.006 | 0.024 |
| Note: ORs and 95% CIs of each allele combinations were calculated with reference to frequencies of all others using Fisher’s exact test. *P* value by Fisher’s exact test. RPL = recurrent pregnancy loss; AOR = adjusted odds ratio; CI = confidence interval. ^a^Fisher’s exact test; ^b^FDR-adjusted *P* value. | | | | | | | | | | | | | | |
