# Supplementary material for: Genetic Variants in MicroRNA Machinery Genes Are Associate with Idiopathic Recurrent Pregnancy Loss Risk
Source: PLoS One. 2014 Apr 25;9(4):e95803. doi: 10.1371/journal.pone.0095803 (PMC4000197; doi:10.1371/journal.pone.0095803)
Supplement: Table S5 — Power calculation to detect an association between a single gene and recurrent pregnancy loss (RPL) with a two-tailed significance level of 0.05 under a case-control design. (DOCX) [file pone.0095803.s005.docx]

**Table S5. Power calculation to detect an association between a single gene and recurrent pregnancy loss (RPL) with a two-tailed significance level of 0.05 under a case-control design**

| **Sample size** | **Gene** | **Minor allele frequency** | **OR** | **Estimated power** |
| --- | --- | --- | --- | --- |
| RPL patients = 338; Controls = 238 | *DICER* rs3742330 | 0.4 | 0.5 | 0.969 |
|  |  |  | 0.6 | 0.818 |
|  |  |  | 0.7 | 0.527 |
|  |  |  | 1.5 | 0.663 |
|  |  |  | 1.6 | 0.789 |
|  |  |  | 1.7 | 0.878 |
|  | *DROSHA* rs10719 | 0.3 | 0.5 | 0.934 |
|  |  |  | 0.6 | 0.745 |
|  |  |  | 0.7 | 0.462 |
|  |  |  | 1.5 | 0.626 |
|  |  |  | 1.6 | 0.756 |
|  |  |  | 1.7 | 0.852 |
|  | *RAN* rs14035 | 0.2 | 0.4 | 0.962 |
|  |  |  | 0.5 | 0.838 |
|  |  |  | 0.6 | 0.613 |
|  |  |  | 1.7 | 0.765 |
|  |  |  | 1.8 | 0.849 |
|  |  |  | 1.9 | 0.901 |
|  | *XPO5* rs11077 | 0.1 | 0.3 | 0.916 |
|  |  |  | 0.4 | 0.781 |
|  |  |  | 0.5 | 0.589 |
|  |  |  | 1.9 | 0.723 |
|  |  |  | 2.0 | 0.800 |
|  |  |  | 2.1 | 0.854 |

Note: RPL = recurrent pregnancy loss; OR = odds ratio
